# Effects of Lutein and Zeaxanthin on LPS-Induced Secretion of IL-8 by Uveal Melanocytes and Relevant Signal Pathways

**DOI:** 10.1155/2015/152854

**Published:** 2015-11-02

**Authors:** Shih-Chun Chao, Tommaso Vagaggini, Chan-Wei Nien, Sheng-Chieh Huang, Hung-Yu Lin

**Affiliations:** ^1^Department of Ophthalmology, Show Chwan Memorial Hospital, No. 526, Sec. 1, Zhongshan Road, Changhua 500, Taiwan; ^2^Institute of Electrical and Computer Engineering, National Chiao Tung University, Hsinchu 30010, Taiwan; ^3^Central Taiwan University of Science and Technology, No. 666, Buzih Road, Beitun District, Taichung 40601, Taiwan; ^4^Albert Einstein College of Medicine, Yeshiva University, 1300 Morris Park Avenue, Bronx, NY 10461, USA; ^5^Department of Optometry, Yuan Pei University, Hsinchu 30015, Taiwan

## Abstract

The effects of lutein and zeaxanthin on lipopolysaccharide- (LPS-) induced secretion of IL-8 by uveal melanocytes (UM) were tested in cultured human UM. MTT assay revealed that LPS (0.01–1 *μ*g/mL) and lutein and zeaxanthin (1–10 *μ*M) did not influence the cell viability of cultured UM. LPS caused a dose-dependent increase of secretion of IL-8 by cultured UM. Lutein and zeaxanthin did not affect the constitutive secretion of IL-8. However, lutein and zeaxanthin decreased LPS-induced secretion of IL-8 in cultured UM in a dose-dependent manner. LPS significantly increased NF-*κ*B levels in cell nuclear extracts and p-JNK levels in the cell lysates from UM, but not p-p38 MAPK and p-ERG. Lutein or zeaxanthin significantly reduced LPS-induced increase of NF-*κ*B and p-JNK levels, but not p38 MAPK and ERG levels. The present study demonstrated that lutein and zeaxanthin inhibited LPS-induced secretion of IL-8 in cultured UM via JNK and NF-*κ*B signal pathways. The anti-inflammatory effects of lutein and zeaxanthin might be explored as a therapeutic approach in the management of uveitis and other inflammatory diseases of the eye.

## 1. Introduction

Zeaxanthin and lutein are two natural bioactives that belong to the xanthophyll class. They are present in the eye in high concentrations. The retina and lens in general and the macular region in the center of the retina in particular are highly enriched in these two xanthophylls [1, 2]. There is increasing evidence that lutein and zeaxanthin may play an important role in protecting against several eye diseases, such as age-related macular degeneration (AMD) [3–8]. Therefore, lutein and zeaxanthin are widely used as nutrient supplements for the prevention and treatment of AMD and other eye diseases. The protective effects of lutein and zeaxanthin may be related to their short wave light-screening effect and antioxidant properties [1–9]. Recent studies indicated that they also influence cell function through various signal pathways or transcription factors and have anti-inflammatory effect [9, 10]. It has been reported that lutein can suppress the development of uveitis caused by injection of lipopolysaccharide (LPS) in rats and mice [10, 11].

LPS is an endotoxin that can induce a potent inflammatory response [12]. Injection of LPS into mice or rats can generate endotoxin-induced uveitis (EIU), which is an important experimental uveitis model in animals [10–16]. LPS injection causes significant increase of several cytokines and chemokines in the eye [10]. Interleukin-8 (IL-8)/CXCL8, a proinflammatory chemokine, plays an important role in the pathogenesis of LPS-induced uveitis. Injection of anti-IL-8 antibodies can block LPS-induced uveitis [15].

Animal studies indicated that lutein reduces the secretion of proinflammatory cytokines and chemokines and this may be the mechanism of inhibition of LPS-induced uveitis by lutein [10]. However, the cell type and the signal pathways involved in this process remain to be studied.

Uveal melanocytes (UM) are the predominant cell type in the uvea. In the past, very little was known about the function of UM and the role of UM in the pathogenesis of various eye diseases. In the past decades, after the development of methods for the culture of UM and the establishment of in vitro models for studying the function of UM, it has been reported that UM produce various growth factors, cytokines, and chemokines, in addition to their functions related to melanin [17–19]. Hu et al. reported that UM produce IL-8 constitutively and the secretion of IL-8 could be increased significantly by the stimulation of LPS [19]. This suggested that UM might play a role in the pathogenesis of ocular inflammatory diseases and may be involved in the inhibitory effects of lutein on LPS-induced uveitis. However, the effects of lutein and zeaxanthin on the secretion of chemokines by UM have not been reported previously. The purposes of this study were to investigate the effects of lutein and zeaxanthin on LPS-induced secretion of IL-8 in human UM in vitro and to study the signal pathways involved in this process.

## 2. Material and Methods

### 2.1. Reagents

Cell culture medium, fetal bovine serum, and trypsin were obtained from GIBCO (Grand Island, NY, USA). LPS, dimethyl sulfoxide (DMSO), collagenase, lutein, 3-[4,5-dimethylthiazol-2-yl]-2,5-diphenyltetrazolium bromide (MTT), phosphate buffered saline (PBF), and protease inhibitor cocktail were obtained from Sigma (St. Louis, MO, USA). Zeaxanthin was obtained from ZeaVision LLC (Chesterfield, MO, USA). Quantikine IL-8 ELISA kit was obtained from R&D System (Minneapolis, MN, USA). p38 mitogen-activated protein kinase (MAPK), extracellular signal-regulated kinases 1/2 (ERK1/2), and c-Jun N-terminal kinase 1/2 (JNK1/2) ELISA kits and cell extraction buffer, PMSF, hypotonic buffer, and nuclear factor-kappa B (NF-*κ*B) ELISA kits were obtained from Invitrogen (Carlsbad, CA, USA).

### 2.2. Cell Culture

Human UM were isolated from donor eyes by trypsin-collagenase sequential method and cultured in FIC medium as previously reported [17–19]. Cells from a primary culture of melanocytes isolated from choroid at passage levels 3–5 were used in this study.

### 2.3. Cell Viability Assay

MTT assay was used to determine the effects of LPS, lutein, and zeaxanthin on cell viability of cultured UM as previously described [19]. For each experiment, 5 × 10^3^ cells were seeded into each well in 96-well plates. After incubation for 24 h, LPS, lutein, or zeaxanthin at different levels was added. Twenty-four hours later, 50 *μ*L of 1 mg/mL MTT was added and culture plates were incubated at 37°C for 4 h. The medium was removed and 100 *μ*L of DMSO was added. The optical density as the parameter of cell viability was measured at 540 nm with a microplate reader (Multiskan EX, Thermo, Ventana, Finland).

### 2.4. LPS and Secretion of IL-8 by UM

Human UM (1 × 10^5^ cells/well) were seeded into the 24-well plates and cultured. When cells reached 80–90% confluence, they were rinsed with PBS and incubated in serum-free culture medium with or without LPS. After 24 h, the conditioned media were collected and centrifuged. The supernatants were stored at −80°C.

### 2.5. Lutein and Zeaxanthinon Constitutive Secretion of IL-8 by UM

Human UM (1 × 10^5^ cells/well) were seeded into the 24-well plates and cultured. When cells reached 80–90% confluence, they were rinsed with PBS and incubated in serum-free culture medium with or without lutein or zeaxanthin. After 24 h, the conditioned media were collected and centrifuged. The supernatants were stored at −80°C.

### 2.6. Lutein and Zeaxanthin on LPS-Induced Secretion of IL-8 by UM

Human UM (1 × 10^5^ cells/well) were seeded into the 24-well plates and cultured, and culture medium was replaced as described above. Lutein or zeaxanthin at different final levels was added. Two hours later, LPS at the final levels of 0.1 *μ*g/mL was added to the cultures. After 24 h, the conditioned media were collected and centrifuged. The supernatants were stored at −80°C.

### 2.7. Measurement of IL-8 Protein Levels

Enzyme-linked immunosorbent assay (ELISA) was used for the measurement of IL-8 protein levels in the supernatant of cultured cells. A commercially available Quantikine IL-8 ELISA kit was used to determine IL-8 protein levels according to the protocol provided by the manufacturer. The optical density of the ELISA samples was measured at 450 and 540 nm using a microplate reader. The sensitivity of the assay for IL-8 was 3.5 pg/mL.

### 2.8. LPS, Lutein, and Zeaxanthin on MAPK and NF-*κ*B

For the study of phosphorylated- (p-) p38 MAPK, p-ERK1/2, and p-JNK1/2 levels, human cultured UM (1 × 10^6^ cells/well) were seeded into 6-well plates and cultured for 24 h and culture medium was replaced as described above. Lutein or zeaxanthin at different final levels was added. Two hours later, LPS at the final levels of 0.1 *μ*g/mL was added into cultures and cultured for 60 min. The cultured media were removed and the cultures were washed with PBS. UM were harvested by scraping with a rubber policeman. Cell were washed with PBS and centrifuged. Cell extraction buffer with protease inhibitor cocktail and PMSF were added to the pellets and cultured for 30 min and centrifuged. All of these procedures were done under 4°C. The supernatants were collected and stored at −80°C. For the study of NF-*κ*B, cells were cultured, treated with or without lutein, zeaxanthin, and LPS, and collected as described above. Collected cells were treated with hypotonic buffer and centrifuged. The pellets that contain nuclear fraction were collected, treated with cell extraction buffer, vortexed, and centrifuged. The supernatants were stored at −80°C.

### 2.9. MAPK and NF-*κ*B Assay

ELISA was used for the measurement of MAPK and NF-*κ*B. Commercially available p38 MAPK, ERK1/2, and JNK1/2 ELISA kits were used to determine p-p38 MAPK, p-ERK1/2, and p-JNK1/2 levels in cell extracts, respectively. The test was performed according to the protocol provided by the manufacturer. The sensitivity of these kits was 0.8 U/mL. NF-*κ*B ELISA kits were used to determine NF-*κ*B levels in cell nuclear extracts according to the protocol provided by the manufacturer. The sensitivity of this kit was <50 pg/mL.

### 2.10. Statistical Analysis

Each experiment was replicated 3 times and the data were presented as mean ± standard deviation (SD). A one-way analysis of variance (ANOVA) test was performed to assess the significance. Values of *p* < 0.05 were considered statistically significant. All data analysis was performed using specific software (SPSS 19.0, SPSS Inc., Chicago, IL, USA).

## 3. Results

### 3.1. Cell Viability Assay

**Figure 1:**
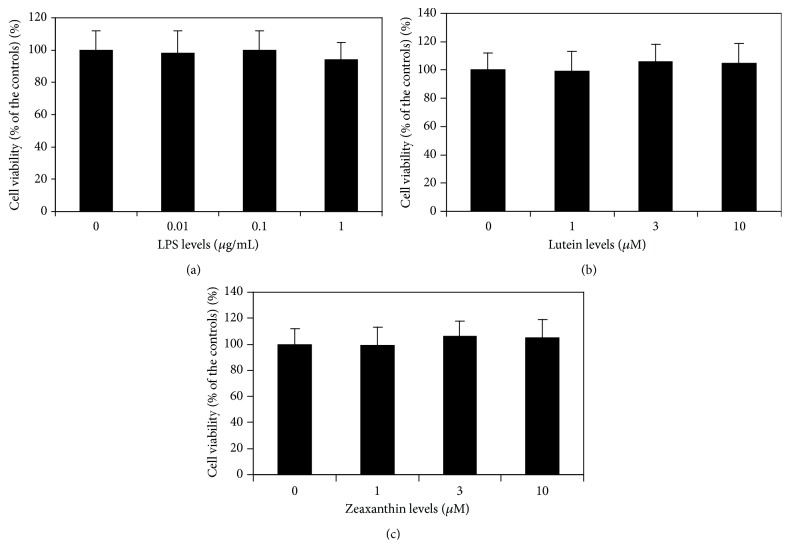
Effects of LPS, lutein, and zeaxanthin on viability of uveal melanocytes (UM). Cells were seeded into 96-well plates and treated with LPS, lutein, and zeaxanthin at different levels. Cell viability was determined by MTT assay (see Material and Methods). LPS (a), lutein (b), and zeaxanthin (c) at all tested levels did not affect the viability of UM (expressed as percentage of the controls) (*p* > 0.05).

MTT assay showed that LPS at the final levels of 0.01, 0.1, and 1 *μ*g/mL did not influence the cell viability of cultured human UM (*p* > 0.05, compared with cells not treated with LPS) (Figure 1(a)). Lutein and zeaxanthin at the final levels of 1, 3, and 10 *μ*M also had no effects on the cell viability of cultured UM (*p* > 0.05, compared with cells not treated with LPS) (Figures 1(b) and 1(c)). Therefore, level ranges of 0.01–1 *μ*g/mL of LPS and 1–10 *μ*M of lutein and zeaxanthin were chosen for subsequent experiments.

### 3.2. LPS and Secretion of IL-8 by UM

**Figure 2:**
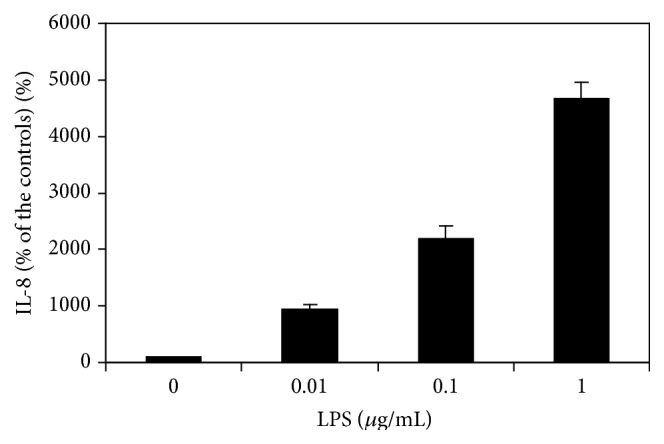
LPS stimulates secretion of IL-8 by uveal melanocytes. Cells were plated into 24-well plates and treated with LPS at different levels. IL-8 protein levels in the conditioned medium were determined by ELISA kit and expressed as percentage of the controls. LPS significantly increased the secretion of IL-8 dose-dependently (*p* < 0.05).

ELISA analysis of cell supernatants of UM cultured with serum-free culture medium detected a low level of IL-8 protein (9.20 ± 0.90 pg/mL), indicating a low level of constitutive secretion of cultured UM. LPS at 0.01–1.0 *μ*g/mL caused a dose-dependent significant increase of IL-8 levels (*p* < 0.05 at all levels of LPS as compared with cells not treated with LPS, Figure 2).

### 3.3. Lutein and Zeaxanthin on Constitutive Secretion of IL-8 by UM

**Figure 3:**
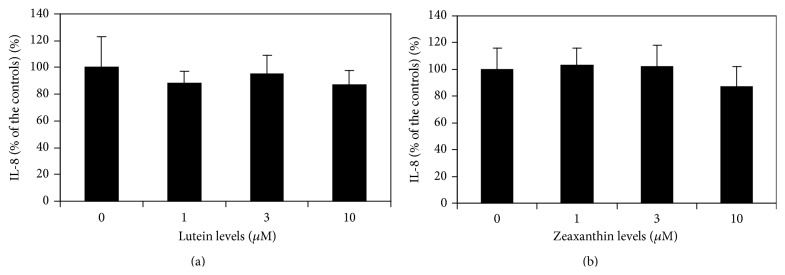
Lutein and zeaxanthinon constitutive secretion of IL-8 by uveal melanocytes. Cells were plated into 24-well plates and treated with lutein or zeaxanthin at different levels. IL-8 protein levels (expressed as percentage of the controls) in cell supernatants from cells treated with lutein (a) or zeaxanthin (b) at different levels did not significantly differ from those from cells not treated with lutein and zeaxanthin (*p* > 0.05).

IL-8 protein levels in cell supernatants from cells treated with lutein or zeaxanthin (1, 3, and 10 *μ*M) did not significantly differ from those from cells not treated with lutein and zeaxanthin (*p* > 0.05, Figure 3), suggesting that lutein and zeaxanthin do not affect the constitutive secretion of IL-8 from UM.

### 3.4. Lutein and Zeaxanthin on LPS-Induced Secretion of IL-8 by UM

**Figure 4:**
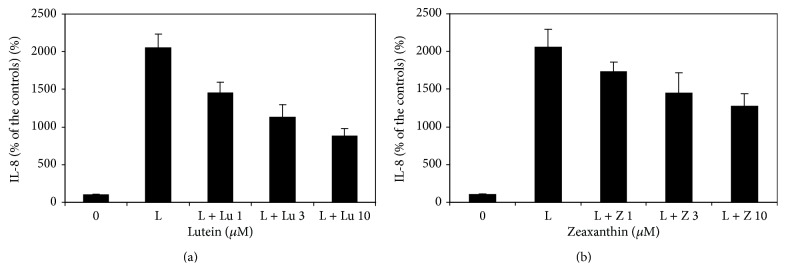
Lutein and zeaxanthin on LPS-induced secretion of IL-8 by uveal melanocytes (UM). Cells were plated into 24-well plates and treated with lutein or zeaxanthin, followed by addition of LPS (0.1 *μ*g/mL) or not. Lutein (a) and zeaxanthin (b) dose-dependently decreased LPS-induced secretion of IL-8 in cultured UM (*p* < 0.05 as compared with cells treated by 0.1 *μ*g/mL LPS only). IL-8 levels in cells treated with lutein or zeaxanthin and LPS were still greater than those from cells not treated with LPS (*p* < 0.05), suggesting that both xanthophylls have a partial inhibitory effect on LPS-induced secretion of IL-8.

Lutein (1, 3, and 10 *μ*M) dose-dependently decreased LPS-induced secretion of IL-8 in cultured UM (*p* < 0.05 as compared with cells treated by 0.1 *μ*g/mL LPS only, Figure 4(a)). However, IL-8 levels in cells treated with lutein and LPS were still greater than those from cells not treated with LPS (*p* < 0.05), suggesting that lutein has a partial inhibitory effect on LPS-induced secretion of IL-8. Zeaxanthin (1, 3, and 10 *μ*M) dose-dependently decreased LPS-induced secretion of IL-8 in cultured UM (*p* > 0.05, zeaxanthin 1 *μ*M versus the controls and *p* < 0.05, zeaxanthin 3 and 10 *μ*M versus the controls, Figure 4(b)). IL-8 levels in cells treated with zeaxanthin and LPS were still greater than those from cells not treated with LPS (*p* < 0.05), suggesting that zeaxanthin also has a partial inhibitory effect on LPS-induced secretion of IL-8.

### 3.5. LPS, Lutein, and Zeaxanthin on MAPK and NF-*κ*B Pathways

**Figure 5:**
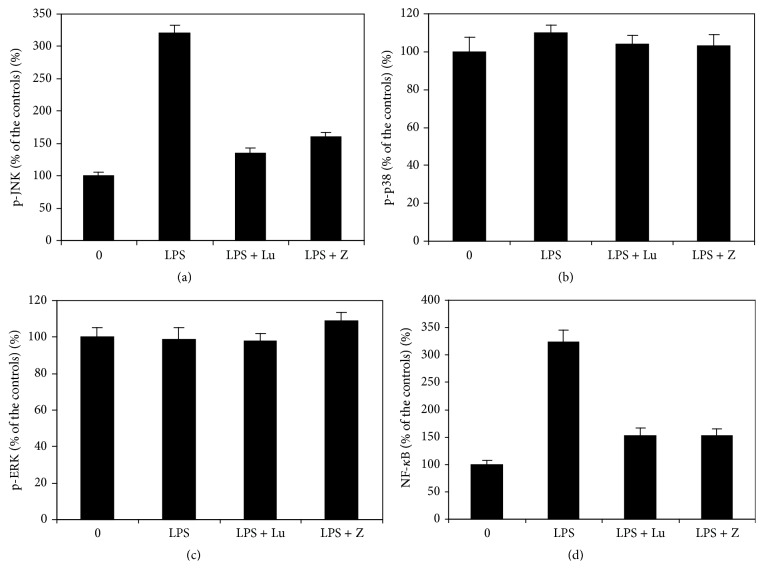
LPS, lutein, and zeaxanthin and various signal pathways. UM were seeded into the culture dishes, treated with LPS with or without previous treatment of lutein or zeaxanthin. p-MAPKs in the cells and NF-*κ*B levels in the cell nuclear extracts were determined by ELISA kit and expressed as percentage of the controls. LPS significantly increased p-JNK (a) and NF-*κ*B levels (d) (*p* < 0.05 as compared with cells not treated with LPS) but not p-p38 MAPK (c) and p-ERG1/2 levels (d) in cultured UM.

LPS at 0.1 *μ*g/mL level significantly increased p-JNK levels (*p* < 0.05 as compared with cells not treated with LPS; Figure 5(a)) but not p-p38 MAPK and p-ERG1/2 levels in cultured UM (*p* > 0.05, Figures 5(b) and 5(c)). Addition of lutein or zeaxanthin significantly reduced LPS-induced increase of p-JNK levels (both *p* < 0.05, Figure 5(a)) but did not affect p-p38 MAPK and p-ERG1/2 levels (both *p* > 0.05, Figures 5(b) and 5(c)) in cultured UM. NF-*κ*B levels in cell nuclear extracts from cells treated with LPS were significantly greater than those from cells not treated with LPS (*p* < 0.05, Figure 5(d)). Lutein or zeaxanthin significantly reduced LPS-induced increase of NF-*κ*B levels in cell nuclear extracts (both *p* < 0.05, Figure 5(d)). These results suggested that JNK1/2 and NF-*κ*B (but not p38 MAPK and ERK1/2) play an important role in LPS-induced increased secretion of IL-8 and in the inhibitory effects of lutein and zeaxanthin on LPS-induced increased secretion of IL-8.

## 4. Discussion

In the present study, we demonstrated that lutein and zeaxanthin inhibited LPS-induced secretion of IL-8 in cultured human UM and this effect was mediated by JNK1/2 and NF-*κ*B signaling pathways.

Uveitis is a common eye disease and a major cause of visual impairment throughout the world [10, 11, 20]. Intraocular or systemic injection of LPS can induce uveitis in experimental animals (EIU). EIU is a well-known model of experimental uveitis used for the study of human uveitis [10–16, 19].

LPS is an endotoxin and is the major component of the outer membrane of Gram-negative bacteria. LPS can induce a strong response from the immune system [12]. LPE-induced uveitis is thought to be the result of a cytokine-chemokine cascade [10, 14]. IL-8 is a critical chemokine in the development and regulation of EIU. IL-8 levels increased significantly in LPS-induced uveitis [15, 16]. Intraocular injection of anti-IL-8 antibody inhibits leukocyte accumulation and decreases the clinical and histological grades of inflammation in LPE-induce uveitis [15]. In vitro studies suggested that LPS induces expression of IL-8 in various cell types [21–23].

Chemokines act as chemoattractants and activators of specific leukocytes at the site of inflammation. Chemokines could be classified into four subfamilies based on the number and location of the cysteine residues at the N-terminus of the molecule and are named CC (with two adjacent cysteines near the N-terminus of the molecule), CXC (the two cysteines being separated by an amino acid), CX3C (having three amino acids between the two cysteines), and C (having a specific amino acid sequence of glutamic acid-leucine-arginine immediately before the first cysteine), in agreement with the systematic nomenclature. In the two main subfamilies (CC and CXC), CXC chemokines are important in the attraction of neutrophils and CC chemokines have powerful chemoattractants and activators for monocytes and lymphocytes [19, 21, 22]. IL-8 is a prototype of CXC chemokine family and is a potent stimulus for neutrophils recruitment and activation. It also triggers the migration and adhesion of T cells, monocytes, and basophils to vascular endothelium and leads to extravasation of these cells into the tissues [19, 21]. Biological activities of IL-8 are mediated by two cell surface G-protein-coupled receptors, CXCR1 and CXCR2 [21, 22]. IL-8 acts as a proinflammatory chemokine and plays an important role in the pathogenesis of the inflammatory process [21, 22]. In the eye, intraocular injection of IL-8 induces uveitis in experimental animals [24]. IL-8 levels are significantly increased in the aqueous humor or vitreous from patients with various types of uveitis [19, 20, 25, 26].

It has been reported that intravenous injection or oral administration of lutein suppresses the development of LPE-induced uveitis in rats and mice, respectively [10, 11]. The anti-inflammatory effect of 100 mg/kg lutein was as strong as that of 1 mg/kg dexamethasone [10]. During the development of EIU, various inflammatory factors significantly increased in the aqueous humor [10]. Lutein significantly decreased the levels of these inflammatory factors in the aqueous humor. The mechanism of the anti-inflammatory effect of lutein may be related to the suppression of proinflammation signal pathways [10]. In addition to the anti-inflammatory effects, lutein has neuroprotective effects on retinal neurons during experimental uveitis and retinitis caused by LPS [27].

In the present study, LPS significantly induced the secretion of IL-8 by UM in a dose-dependent manner, which is consistent with previous reports [19]. Lutein and zeaxanthin dose-dependently inhibit LPS-induced increased secretion of IL-8 in cultured UM; this is consistent with the animal studies, which showed that lutein suppresses the occurrence of LPE-induced uveitis [10]. To the best of our knowledge, this is the first report showing that lutein and zeaxanthin inhibit LPS-induced expression of IL-8 in UM.

The mechanism of lutein and zeaxanthin inhibition of LPS-induced expression of chemokines in UM has not been previously reported. The present study demonstrated that lutein and zeaxanthin inhibited the secretion of IL-8 induced by LPS through the activation of JNK1/2 and NF-*κ*B signal pathway, but not p38 and ERK pathway; this is consistent with the animal study, which showed that lutein inhibited the activation of NF-*κ*B in the iris-ciliary body in LPS-induced uveitis and in cultured macrophages [9, 10].

In conclusion, this study demonstrated that lutein and zeaxanthin inhibited LPS-induced secretion of IL-8 in cultured human UM and this effect was mediated by JNK1/2 and NF-*κ*B signaling pathways. Inhibition of secretion of IL-8 by lutein and zeaxanthin might be explored as a therapeutic approach in the management of uveitis and other inflammatory diseases of the eye.
